# Postsurgical Pain Risk Stratification to Enhance Pain Management Workflow in Adult Patients: Design, Implementation, and Pilot Evaluation

**DOI:** 10.2196/54926

**Published:** 2024-07-02

**Authors:** Matthias Görges, Jonath Sujan, Nicholas C West, Rama Syamala Sreepada, Michael D Wood, Beth A Payne, Swati Shetty, Jean P Gelinas, Ainsley M Sutherland

**Affiliations:** 1 Department of Anesthesiology, Pharmacology & Therapeutics The University of British Columbia Vancouver, BC Canada; 2 Research Institute BC Children’s Hospital Vancouver, BC Canada; 3 School of Population and Public Health The University of British Columbia Vancouver, BC Canada; 4 MD Undergraduate Program The University of British Columbia Vancouver, BC Canada; 5 Department of Anesthesiology & Pain Medicine University of Alberta Edmonton, AB Canada; 6 Department of Anesthesiology Surrey Memorial Hospital Surrey, BC Canada; 7 Department of Anesthesiology St. Paul’s Hospital Vancouver, BC Canada

**Keywords:** patient-oriented research, patient-reported outcome measures, risk prediction, pain, individualized risk, surgery, anesthesia, opioid analgesia, short-term opioid use, care planning, digital health platforms

## Abstract

**Background:**

Exposure to opioids after surgery is the initial contact for some people who develop chronic opioid use disorder. Hence, effective postoperative pain management, with less reliance on opioids, is critical. The Perioperative Opioid Quality Improvement (POQI) program developed (1) a digital health platform leveraging patient-survey-reported risk factors and (2) a postsurgical pain risk stratification algorithm to personalize perioperative care by integrating several commercially available digital health solutions into a combined platform. Development was reduced in scope by the COVID-19 pandemic.

**Objective:**

This pilot study aims to assess the screening performance of the risk algorithm, quantify the use of the POQI platform, and evaluate clinicians’ and patients’ perceptions of its utility and benefit.

**Methods:**

A POQI platform prototype was implemented in a quality improvement initiative at a Canadian tertiary care center and evaluated from January to September 2022. After surgical booking, a preliminary risk stratification algorithm was applied to health history questionnaire responses. The estimated risk guided the patient assignment to a care pathway based on low or high risk for persistent pain and opioid use. Demographic, procedural, and medication administration data were extracted retrospectively from the electronic medical record. Postoperative inpatient opioid use of >90 morphine milligram equivalents per day was the outcome used to assess algorithm performance. Data were summarized and compared between the low- and high-risk groups. POQI use was assessed by completed surveys on postoperative days 7, 14, 30, 60, 90, and 120. Semistructured patient and clinician interviews provided qualitative feedback on the platform.

**Results:**

Overall, 276 eligible patients were admitted for colorectal procedures. The risk algorithm stratified 203 (73.6%) as the low-risk group and 73 (26.4%) as the high-risk group. Among the 214 (77.5%) patients with available data, high-risk patients were younger than low-risk patients (age: median 53, IQR 40-65 years, vs median 59, IQR 49-69 years, median difference five years, 95% CI 1-9; *P*=.02) and were more often female patients (45/73, 62% vs 80/203, 39.4%; odds ratio 2.5, 95% CI 1.4-4.5; *P*=.002). The risk stratification was reasonably specific (true negative rate=144/200, 72%) but not sensitive (true positive rate=10/31, 32%). Only 39.7% (85/214) patients completed any postoperative quality of recovery questionnaires (only 14, 6.5% patients beyond 60 days after surgery), and 22.9% (49/214) completed a postdischarge medication survey. Interviewed participants welcomed the initiative but noted usability issues and poor platform education.

**Conclusions:**

An initial POQI platform prototype was deployed operationally; the risk algorithm had reasonable specificity but poor sensitivity. There was a significant loss to follow-up in postdischarge survey completion. Clinicians and patients appreciated the potential impact of preemptively addressing opioid exposure but expressed shortcomings in the platform’s design and implementation. Iterative platform redesign with additional features and reevaluation are required before broader implementation.

## Introduction

### Background

The ongoing opioid overdose epidemic has contributed to unprecedented and unnecessary deaths, with an estimated 100,306 deaths from prescription and illegal opioid use in the United States in the 12 months before April 2021 [1] and 5360 deaths in Canada in the first 9 months of 2022 [2]. For many patients with an opioid use disorder, the perioperative period represents the source of initial exposure (>6% compared to 0.4% in a control cohort without surgery in the United States) [3]. Hence, effective postoperative pain management, with less reliance on the prescription of opioids, could be a valuable mechanism to reduce the development of subsequent opioid use disorder. Postsurgical opioids are most frequently prescribed by the surgeon and followed up by the patient’s primary care physician [4]. Anesthesiologists are uniquely positioned to manage acute postoperative pain effectively with multimodal analgesia to decrease perioperative opioid exposure and prevent subsequent persistent opioid use [3].

Perioperative health care is being optimized through enhanced recovery after surgery (ERAS) pathways [4-6], multimodal analgesic plans [5,7,8], and regional anesthesia techniques [9]. Further opportunities to improve postsurgical pain trajectories are offered by prehabilitation programs [10-12], our developing understanding of the risks of persistent postsurgical pain [13-17], and the feasibility of accessing and analyzing large volumes of data. A critical step is identifying patients at high risk of significant postsurgical pain and long-term opioid use.

The Perioperative Opioid Quality Improvement (POQI) program was designed to address the ongoing opioid use epidemic in British Columbia, where opioid use disorder continues to be one of the most pressing public health concerns. Recent studies have highlighted the scale of the local opioid problem and highlighted the case for addressing opioid risk during routine clinical care, including surgery: 12% of our population received an opioid prescription in 2017, with the number of people who receive a high dose (>90 morphine milligram equivalents [MME]/day) increasing during the period from 2013 to 2017 [18]; patients with opioid overdose have often had previous clinical encounters for pain (50%) and surgery (5%) [19].

The POQI program was funded in 2019 by DIGITAL, Canada’s Global Innovation Cluster for digital technologies, as a consortium between digital health companies, health care organizations, and university partners. It aimed to develop and implement a postsurgical pain risk stratification algorithm by integrating several commercially available digital health solutions into a combined POQI digital health platform for prehabilitation and postsurgical care planning. The COVID-19 pandemic adversely impacted the ability to engage clinicians and patients in co-designing and testing the solution iteratively. Hence, the project faced significant delays, and the scope of the POQI platform development was reduced. Specifically, planned features for 2-way communication and personalization of educational information for patients were not included in the prototype tested in this study.

### Objectives

The specific objectives of the pilot deployment of the POQI platform were to assess (1) the screening performance of the risk stratification algorithm to facilitate subsequent risk score optimization and (2) the use, utility, and perceived benefit of the POQI platform among end users (clinicians and patients).

## Methods

### Study Design and Approval

The study involved the design, implementation, and pilot evaluation of the POQI digital health platform at Providence Health Care’s (PHC’s) St. Paul’s Hospital in Vancouver, British Columbia, Canada. The target users were clinicians and patients. The patient population for pilot-testing had undergone a designated set of colorectal surgeries; this population was selected because the colorectal surgical clinic was an early adopter of an electronic health history questionnaire (HHQ) upon which the platform expanded. As a result of this initiative, the Department of Anesthesiology and Pain Medicine at PHC established a new Transitional Pain Clinic for patients at risk of persistent postoperative pain or opioid use after surgery. It held weekly clinics during the study period and continued to serve St. Paul’s Hospital patients after the study concluded.

The POQI platform incorporated an algorithm [20] that classified patients as low risk or high risk for persistent postsurgical pain and long-term opioid use. Clinicians used this classification to assign patients to low-risk or high-risk pathways for personalized prehabilitation, patient education, and care planning. Specifically, patients were told that there were resources that they could use to learn about pain and nonpharmacologic strategies for pain management and that they could keep track of their medication use and pain scores over time in the system. The performance of this risk stratification was evaluated based on observed postoperative inpatient opioid use. The clinician and patient user experiences were evaluated using mixed methods.

### Ethical Considerations

The University of British Columbia PHC Research Ethics Board determined this work to be a quality improvement project (reviewed on October 13, 2020), for which they do not require ethical review under Article 2.5 of the Canadian Tri-Council Policy Statement [21]. Hence, this project was run as a quality improvement pilot project governed by Privacy Impact Assessment and Security Threat and Risk Assessment. This manuscript adheres to the SQUIRE 2.0 (Standards for Quality Improvement Reporting Excellence) reporting guidelines [22].

### The POQI Digital Health Platform

**Figure 1:**
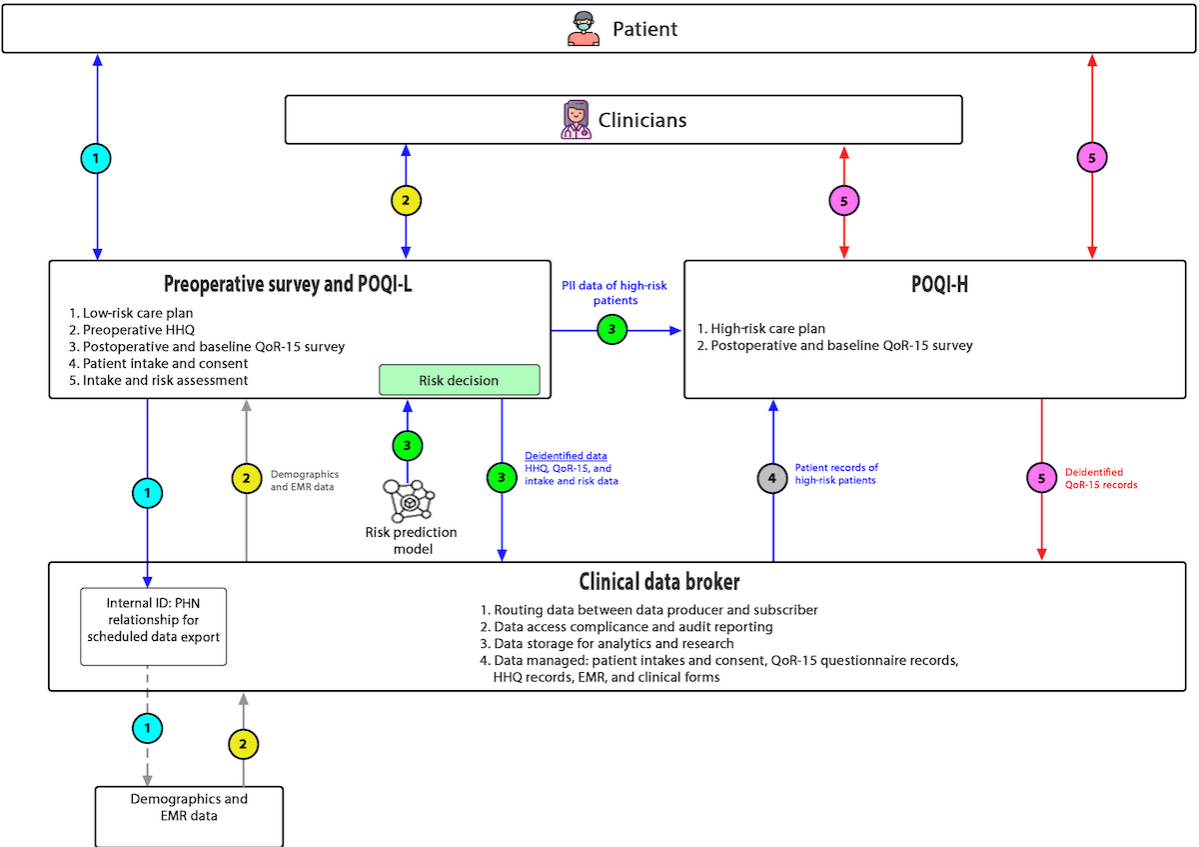
Workflow in the perioperative quality improvement (POQI) platform showing the integration of clinical and patient-reported data from patient-facing components and the electronic medical record (EMR) integrated by a data broker. PHN: personal health number; PII: personally identifiable information; POQI-H: POQI platform for high-risk patients; POQI-L: POQI platform for low-risk patients; QoR: quality of recovery.

Development of the POQI platform combined existing technologies from 3 industry partners (Figure 1): a preoperative survey and POQI platform for low-risk patients (POQI-L), supplied by Thrive Health; a POQI platform for high-risk patients (POQI-H), supplied by Careteam Technologies; and a data broker, supplied by Excelar Technologies (also incorporating Xerus Medical from 2021). Additional components were identified and developed based on the needs of the clinical implementation partners (the anesthesiologists and perioperative care team at St. Paul’s Hospital). The platform’s original scope of development work was scaled back due to resource and time constraints during the COVID-19 pandemic. The resultant POQI platform used in this study should be considered an initial prototype. Original development plans included (1) additional iterations of user testing and design refinement; (2) additional features, such as 2-way communication between patients and clinicians; and (3) personalization of educational materials to meet patients’ needs optimally.

The prototype POQI platform allowed for the collection of patient-specific data, including a presurgical HHQ (questions selected as risk factors for modeling are presented in Multimedia Appendix 1) and patient-reported outcome measures (PROMs) at baseline. Furthermore, data were collected postoperatively using quality of recovery-15 (QoR-15) questionnaires [23] and additional PROM surveys to collect self-reported medication use and pain (scores). The platform was linked to an automated export from the Cerner electronic medical record (EMR) system (Cerner Corp), which allowed for collecting surgery details and oral and intravenous opioid use data from inpatient medication administration records.

Initial HHQ data were used to stratify patients for risk of persistent postsurgical pain and opioid use, using a previously developed risk score, which was based on the data collected from 122 patients who underwent colorectal surgery; 22 (18%) of them had high postoperative opioid use, which was strongly associated with a history of chronic pain, substance use disorder, and open surgery [20]. Patients were categorized into high-risk and low-risk groups using a point-based prediction model that considered 11 risk factors with different weights [20]: substance use disorder (risk score weight=5); current prescription of opioid (risk score weight=5), benzodiazepine (risk score weight=4), or antidepressant (risk score weight=4); recreational drug use (risk score weight=4); history of chronic pain (risk score weight=4), anxiety or panic attacks (risk score weight=2), depression (risk score weight=2), or poorly controlled pain after surgery (risk score weight=2); female sex (risk score weight=2); and age <40 years (risk score weight=1; refer to relevant HHQ questions in Multimedia Appendix 1). The algorithm flagged a patient as high risk if the risk score was >7 out of 35, after which a clinician manually onboarded the patient to the POQI-H platform or confirmed that they should remain on the POQI-L platform. The clinician could override the algorithm’s proposed risk label if they deemed it clinically appropriate. In addition, clinicians could use their clinical judgment to manually onboard patients directly to POQI-H after the St. Paul’s Hospital Transitional Pain Clinic consultation, even when no electronic HHQ data were available.

High-risk patients were given a care plan that provided them with education about pain and opioid management and prompted them to record their medication use and pain scores (refer to the *Study Design and Approval* section for details). Some high-risk patients were also seen preoperatively in St. Paul’s Hospital Transitional Pain Clinic for prehabilitation, education, and pain management planning when the responsible clinician deemed it appropriate. Postoperatively, high-risk patients were flagged by St. Paul’s Hospital Transitional Pain Clinic providers for closer follow-up by the Acute Pain Service clinicians in the hospital.

Regardless of the risk categorization, patients who used a significant quantity of opioids postoperatively (>90 MME) were also followed by St. Paul’s Hospital Transitional Pain Service for optimization of their postdischarge pain management and opioid weaning; 90 MME was chosen as the threshold for referral, as it is recommended in the 2017 Canadian Guideline for Opioids for Chronic Non-Cancer Pain that patients using >90 MME per day be weaned to the lowest effective dose, potentially including discontinuation [24].

### Participants and Recruitment

**Figure 2:**
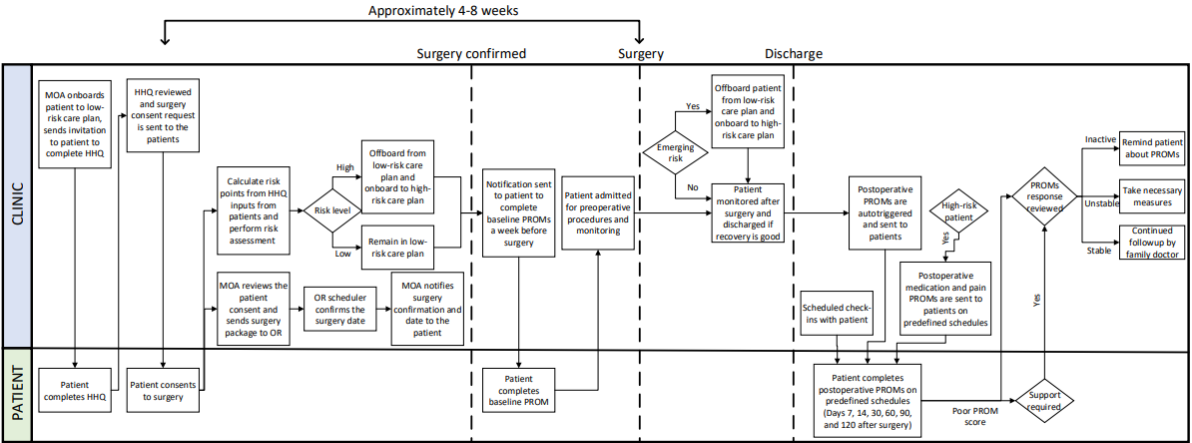
Clinical workflow of the perioperative quality improvement platform as piloted at St. Paul’s Hospital. This figure illustrates the flow of patients through their perioperative care journey and delineates which pieces the system performs and when the patient is involved in this process; it shows key decision points, such as when the patient is risk stratified before their procedure and whether patients require enhanced follow-up after discharge. A poor patient-reported outcome measure (PROM) score (bottom right) was indicated if the patient reported having an unplanned hospital admission for pain, having to seek urgent care for pain, or if they were still taking opioids beyond postoperative day 7. HHQ: health history questionnaire; MOA: medical office assistant; OR: operating room.

Pilot use of the POQI platform was initiated at St. Paul’s Hospital in December 2021 and formally adopted on January 1, 2022. The target population for pilot-testing included patients undergoing a designated set of colorectal surgeries during the active enrollment period (Multimedia Appendix 2) and excluded patients who underwent screening and minimally invasive diagnostic procedures such as endoscopies. Patients who had a surgery that was not included in the designated set or had undergone procedures with a surgical time of <20 minutes were excluded. Furthermore, patients who underwent surgery before January 1, 2022, were excluded, as the complete POQI platform implementation was not available for clinical use until then. Only the surgical encounter closest to the most recently recorded HHQ was considered when patients had multiple procedures. Eligible patients were enrolled for the pilot through routine clinical care by the medical office assistant in surgical clinics (Figure 2). Postoperative data collection continued for up to 120 days after surgery, with surveys potentially completed on postoperative days 7, 14, 30, 60, 90, and 120.

### Data Collection and Management

The patient-specific data, including preoperative baseline HHQ, QoR-15 questionnaires, and PROM surveys, were fed directly to the data broker from the respective POQI-L or POQI-H platforms. The surgery details and opioid use data from the medication administration record were extracted from the EMR. These data were made available in a data lake by the Excelar data broker for analysis. The unifying variables used to link the multiple platforms were the patient’s personal health number and the ThriveID, assigned at the initial onboarding for HHQ completion. Data for this evaluation were aggregated and deidentified (Figure 2). The deidentified data sets were then exported to the research team for analysis.

### Outcomes

#### Risk Stratification

To evaluate the risk stratification, we elected to focus on inpatient opioid use. Analyzing long-term opioid use was not possible: records of opioids dispensed from the provincial medication system (PharmaNet) were not made available due to provincial policy constraints at the time, and patient self-report was deemed to be unfeasible and incomplete or biased. Therefore, the primary outcome used to evaluate the accuracy of the risk stratification was based on inpatient daily opioid use, using a threshold of >90 MME per day to indicate high opioid use, in line with the recommendations for opioid therapy and chronic noncancer pain [24]. MME was computed by multiplying the dosage of opioids delivered to the patient with the MME conversion factor of the corresponding drug and route of administration (Multimedia Appendix 3). For oral methadone, the MME conversion factor varies with the dosage administered per day; consequently, an aggregation algorithm was used to calculate the total methadone administered per day.

Patient-controlled analgesia was typically used for in-hospital intravenous opioid administration. Nurses regularly recorded the number of doses delivered to the patient, and the patient-controlled analgesia pump was reset every 12 hours at the end of their shift. The net amount of drug delivered to the patient was computed using the number of doses and the amount of drug in each dose. The MME values from intravenous and oral administration were then summed for every patient over a 24-hour period, starting at 6 AM and ending at 6 AM the following day.

EMR data structures and export limitations prevented us from including MMEs of drugs delivered through continuous opioid infusion or boluses; these patients were excluded from MME evaluation. Intraoperative opioids were not included when computing MME/day; that is, on the day of surgery, only opioids administered after the surgery up to 6 AM the following day were included for the MME/day calculation.

#### Use, Utility, and Perceived Benefit

The user experience outcomes of use, utility, and perceived benefit were evaluated using mixed methods.

Use was measured quantitatively by evaluating both uptake and attrition with the platform. Uptake was measured by the number of patients completing the HHQ survey and the number completing the preoperative baseline QoR-15. Attrition was evaluated by measuring continued use of the system postoperatively, that is, by the number of patients completing at least 1 postoperative QoR-15 survey, at least 1 PROM survey, and their postoperative data collection period up to the 90-day mark.

Utility and perceived benefit were evaluated through a series of semistructured interviews with both patients and clinicians via Zoom (Zoom Video Communications). To obtain a representative sample, a randomly selected group of 10 patients deemed high risk for significant postsurgical pain and a random group of 10 patients deemed low risk for significant postsurgical pain were contacted approximately 1 week after hospital discharge and invited to participate. For clinicians, we included anesthesiologists and nurses in St. Paul’s Hospital Transitional Pain Clinic and aimed for a sample of 5 clinicians.

Brief (approximately 10-15 minutes) interviews focused on three domains: (1) experience with the platform technologies, (2) perceived benefit of the platform for the health care experience, and (3) feedback or concerns about the platform (Multimedia Appendix 4). Interviews were conducted in a safe environment of mutual respect and facilitated by a medical student (SS) assisting with the project. Transcripts were automatically obtained from Zoom and downloaded from the videoconferencing platform for all interviews. A research team member (MDW) thematically analyzed the transcripts using NVivo (QSR International).

#### Additional Secondary Outcomes

Additional secondary outcomes included emergent readmissions; pain scores over the first 3 postoperative days; and continued opioid use at 30, 60, and 90 days, collected through the additional PROM surveys. To determine the number of patients who had emergent readmissions, we filtered the inpatient and emergency department visit data sets for patients with prior surgery. We confirmed that the admission time in the new visit was after the discharge time following the surgery. As inpatients could have had nonemergent readmissions for scheduled procedures and not all emergent visits require admissions, only the inpatient visits categorized as “urgent/emergent” and the patients admitted after emergency visits were included. The data set was split into readmissions within 30 days and readmissions within 180 days after discharge.

### Statistical Analysis

The available data were summarized for high- and low-risk patients, including patient count, age distribution, surgical wait time (time to surgery after referral for surgical care), procedure duration, length of hospital stay, the identified risk factors from the HHQ (refer to *The POQI Digital Health Platform* section), preoperative and postoperative QoR-15 scores, the proportion of the population that completed the QoR-15, length of follow-up, the number of emergent readmissions, in-hospital opioid use in MME/day, and most prevalent surgeries. Frequency data are reported as n/N (%); the denominator N changes due to data linking issues and loss of follow-up during the study period.

Due to the small sample size, data for low- and high-risk groups were compared using nonparametric statistical tests: the Fisher exact test for counts and the Mann-Whitney *U* test for continuous data. A logistic regression of all risk factors for high in-hospital opioid use was performed to generate adjusted odds ratios (ORs), reported with 95% CIs. Analyses were performed using Python (version 3.10; Python Software Foundation): Pandas (version 1.5.0; Wes McKinney), SciPy (version 1.9.3; Enthought), and NumPy (version 1.23.3) were used for data cleaning, processing, and analysis; Matplotlib (version 3.6.0) was used to generate plots; and Openpyxl (version 3.0.10) was used to create analysis reports. R software (version 4.2.2; The R Foundation) was used for statistical comparisons.

The accuracy of the risk stratification was assessed to determine if the algorithm was sensitive enough to categorize patients based on their health history. This was achieved by constructing confusion matrices using the high- and low-risk labels generated by the risk prediction algorithm (using HHQ data, not POQI-L or POQI-H enrollment labels) and the outcome, that is, high (>90 MME/day) and low (≤90 MME/day) opioid use. These data were used to estimate sensitivity, specificity, false negative rate, false positive rate, and positive and negative likelihoods.

Scatter with line (median) plots and box plots were created to determine the trend of opioid use by patients on postoperative days 0 to 10 and to compare the trend between low- and high-risk patients.

## Results

### Population

**Figure 3:**
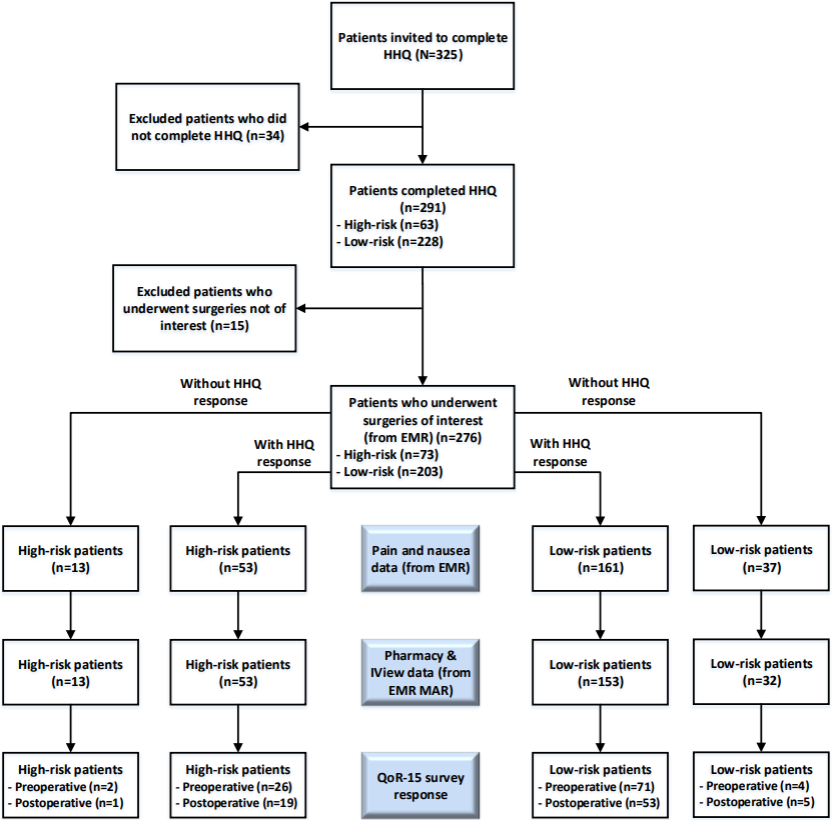
Platform uptake, attrition, and data completeness in high-risk and low-risk patients. EMR: electronic medical record; HHQ: health history questionnaire; MAR: medication administration record; QoR: quality of recovery.

A total of 276 eligible patients were admitted for one of the colorectal procedures selected for inclusion in the study at St. Paul’s Hospital between January 01, 2022, and September 30, 2022, and completed the HHQ before surgery (Figure 3). The denominators vary in the result tables due to the selective completion of surveys and the availability of linked data.

### Risk Stratification Characteristics

Of the 276 patients, the risk stratification algorithm identified 203 (73.6%) patients as low risk and 73 (26.4%) as high risk. The most common surgeries for low-risk patients were laparoscopic resection of the anterior colon, transanal resection of a rectal lesion by assisted microsurgery, and laparoscopic resection of the bowel. The most common surgeries for high-risk patients were laparoscopic resection of the anterior colon, laparoscopic resection of the bowel, and lysis of adhesions.

The most substantial differences in risk factors between the high-risk and low-risk groups were history of depression (OR 29.4, 95% CI 9.2-125; risk score weight=2), antidepressant prescription (OR 23.4, 95% CI 7.9-85.2; risk score weight=4), current opioid prescription (OR 20.4, 95% CI 4.2-196.4; risk score weight=5), and history of chronic pain (OR 19.4, 95% CI 6.9-63.3; risk score weight=4; Table 1).

**Table 1:** Risk factor distribution among cohort and risk groups, with odds ratios for being in the high-risk group. While risk factor details were not available in all cohort patients, the label from the calculation was available.

Risk factor	Total sample (N=214), n (%)	Low-risk group (n=161), n (%)	High-risk group (n=53), n (%)	Odds ratio (95% CI)
Substance use disorder	9 (4.2)	3 (1.9)	6 (11.3)	6.6 (1.4-42.6)
Current opioid prescription	13 (6.1)	2 (1.2)	11 (20.8)	20.4 (4.2-196.4)
Benzodiazepine prescription	9 (4.2)	3 (1.9)	6 (11.3)	6.6 (1.4-42.6)
Antidepressant prescription	28 (13.1)	5 (3.1)	23 (43.4)	23.4 (7.9-85.2)
Recreational drug use	29 (13.6)	10 (6.2)	19 (35.8)	8.3 (3.3-22.0)
History of chronic pain	29 (13.6)	6 (3.7)	23 (43.4)	19.4 (6.9-63.3)
History of anxiety	46 (21.5)	18 (11.2)	28 (52.8)	8.8 (4.0-19.7)
History of depression	27 (12.6)	4 (2.5)	23 (43.4)	29.4 (9.2-125.0)
History of poorly controlled pain	26 (12.1)	11 (6.8)	15 (28.3)	5.3 (2.1-14.0)
Female sex	90 (42.1)	59 (36.6)	31 (58.5)	2.4 (1.2-4.8)
Age (<40 years)	32 (15.0)	20 (12.4)	12 (22.6)	2.1 (0.8-4.9)

High-risk patients were younger than low-risk patients (age: median 53, IQR years, vs median 59, IQR years, median difference [MD] 5 years, 95% CI 1-9; *P*=.02) and were more often female (45/73, 62%, vs 80/203, 39.4%; OR 2.5, 95% CI 1.4-4.5; *P*=.002; Table 2). Furthermore, high-risk patients reported lower baseline (preoperative) QoR scores (median 122, IQR 91-136, vs median 131, IQR 116-140, MD 12, 95% CI 2-23; *P*=.02).

**Table 2:** Preoperative and surgical characteristics of the overall cohort and separate risk groups.

		Total sample (N=276)	Low-risk group (n=203)	High-risk group (n=73)	*P* value		Median difference (95% CI)		Odds ratio (95% CI)	
Age (y), median (IQR)		59 (47-68)	59 (49-69)	53 (40-65)	.02		5 (1 to 9)		—^a^	
**Sex, n (%)**						.002		—		2.5 (1.4 to 4.5)
	Male	151 (54.7)	123 (60.6)	28 (38.4)						
	Female	125 (45.3)	80 (39.4)	45 (61.6)						
**Surgery type, n (%)**						.15		—		1.5 (0.9 to 2.8)
	Closed	183 (66.3)	140 (69.0)	43 (58.9)						
	Open	93 (33.7)	63 (31.0)	30 (41.1)						
Time to surgery (days), median (IQR)^b^		30 (18-68)	29 (16-54)	34 (19-86)	.21		–4.9 (–13.3 to 2.7)		—	
Length of surgery (hours), median (IQR)		2.1 (1.2-3.1)	2.1 (1.1-3.0)	1.9 (1.2-3.3)	.85		0.0 (–0.3 to 0.4)		—	
Preoperative QoR-15^c^ score, median (IQR)^d^		129 (104-139)	131 (116-140)	122 (91-136)	.02		12 (2 to 23)		—	

^a^Not applicable.

^b^Data available: total, n=267; low-risk patients, n=195; high-risk patients, n=75. This indicates the number included in the analysis (eg, surgical decision time is not available for all patients).

^c^QoR-15: quality of recovery-15.

^d^Data available: total, n=110; low-risk patients, n=77; high-risk patients, n=33. This indicates the number included in the analysis.

### Postoperative Outcomes

**Figure 4:**
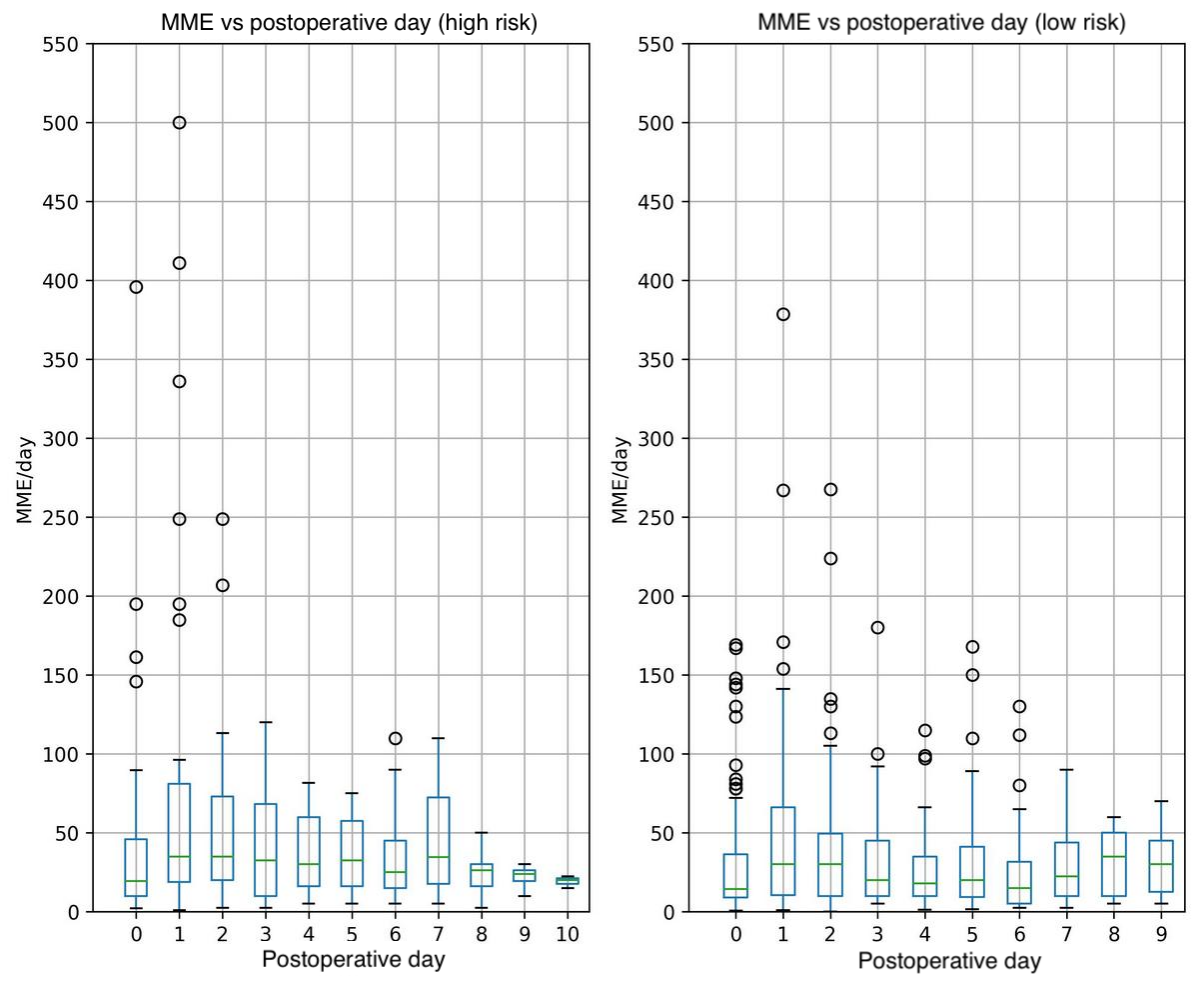
Box plots of morphine milligram equivalents (MME) per day comparing high-risk and low-risk patients.

Overall inpatient opioid use was not significantly different between the 2 risk groups, with a median of 20 IQR (10-45) MME/day in low-risk cases versus a median of 25 IQR (10-50) MME/day in high-risk cases (MD –2, 95% CI –5 to 0; *P*=.10; Table 3). Similarly, no significant difference was observed in opioid use across the recovery profile of low- versus high-risk patients over the first 10 postoperative days (Figure 4). Our risk factors were not strong predictors for high MME/day: none of the ORs from logistic regression were significant (ie, 95% CI range included 1 for all predictors), which differs from our original model building cohort [20] (Table 4, right column).

**Table 3:** Inpatient opioid use in patients with patient-controlled analgesia or oral opioid medications (n=231)^a^.

	Total (n=231)	Low-risk group (n=165)	High-risk group (n=66)	*P* value	Median difference (95% CI)	Odds ratio (95% CI)
MME^b^/day (mg), median (IQR)	24 (10-47)	20 (10-45)	25 (10-50)	.10	–2 (–5 to 0)	—^c^
Total MME (mg), median (IQR)	48 (15-145)	43 (15-130)	65 (18-237)	.09	–10 (–38 to 1)	—
Patients using >90 MME/day, n (%)	31 (13.4)	21 (12.7)	10 (15.1)	.67	—	1.2 (0.5 to 2.9)

^a^Some patients, not included here, had continuous opioid infusion only or no opioid medications.

^b^MME: morphine milligram equivalent.

^c^Not applicable.

**Table 4:** Risk factor distribution among cohort and outcome groups, with the odds ratios for patients using >90 morphine milligram equivalent (MME) per day for which the presurgical health history questionnaire details were available. The adjusted odds ratios from the derivation cohort [20] are provided for reference.

Risk factor	Total sample (n=201), n (%)	≤90 MME/day (n=177), n (%)	>90 MME/day (n=24), n (%)	Unadjusted odds ratio (95% CI)	Adjusted odds ratio (95% CI)^a^	Adjusted odds ratio in the derivation cohort [20] (95% CI)
Substance use disorder	9 (4.5)	7 (4.0)	2 (8.3)	2.2 (0.2-12.6)	1.8 (0.2-9.5)	1.6 (1.0-2.3)
Current opioid prescription	12 (6.0)	9 (5.1)	3 (12.5)	2.6 (0.4-11.8)	2.9 (0.5-12.4)	1.1 (0.7-1.6)
Benzodiazepine prescription	9 (4.5)	8 (4.5)	1 (4.2)	0.9 (0.0-7.4)	0.6 (0.0-4.4)	1.0 (0.8-1.3)
Antidepressant prescription	28 (13.9)	23 (13.0)	5 (20.8)	1.8 (0.5-5.5)	1.6 (0.4-6.2)	1.2 (0.7-1.8)
Recreational drug use	28 (13.9)	25 (14.1)	3 (12.5)	0.9 (0.2-3.2)	0.7 (0.1-2.5)	1.1 (0.6-1.7)
History of chronic pain	28 (13.9)	24 (13.6)	4 (16.7)	1.3 (0.3-4.3)	0.9 (0.2-3.1)	1.6 (1.0-2.6)
History of anxiety	44 (21.9)	35 (19.8)	9 (37.5)	2.4 (0.9-6.5)	2.5 (0.8-7.3)	0.8 (0.5-1.2)
History of depression	26 (12.9)	22 (12.4)	4 (16.7)	1.4 (0.3-4.8)	0.8 (0.2-3.2)	0.9 (0.6-1.3)
History of poorly controlled pain	25 (12.4)	23 (13.0)	2 (8.3)	0.6 (0.1-2.8)	0.5 (0.1-2.1)	1.1 (0.6-1.7)
Female sex	82 (40.8)	72 (40.7)	10 (41.7)	1.0 (0.4-2.7)	0.8 (0.3-2.0)	1.0 (0.6-1.6)
Age (<40 years)	30 (14.9)	26 (14.7)	4 (16.7)	1.2 (0.3-3.9)	1.2 (0.3-4.0)	1.0 (0.9-1.0)
Open surgery	—^b^	—	—	—	—	1.2 (0.7-2.0)

^a^Values derived from multivariate logistic regression, including all other risk factors.

^b^Not applicable.

Readmissions and other postoperative outcomes did not differ between high- and low-risk groups, although the overall median postoperative QoR-15 score was higher in the low-risk group than in the high-risk group (MD 11, 95% CI 4-19; *P*=.002; Table 5).

**Table 5:** Postoperative outcomes.

	Total (n=231)	Low-risk group (n=165)	High-risk group (n=66)	*P* value	Median difference (95% CI)	Odds ratio (95% CI)
Total readmissions, n (%)	75 (32.5)	51 (30.9)	24 (36.4)	.22	—^a^	1.5 (0.8 to 2.7)
Emergent readmissions (within 30 days of surgery), n (%)	20 (8.7)	13 (7.8)	7 (10.6)	.43	—	1.5 (0.5 to 4.4)
Emergent readmissions (30 to 180 days following surgery), n (%)	7 (3.0)	4 (2.4)	3 (4.5)	.39	—	2.1 (0.3 to 12.9)
Length of hospital stay (days), median (IQR)	4 (2-6)	4 (2-6)	5 (1-7)	.56	0 (–1 to 0)	—
Overall postoperative QoR-15^b^ score, median (IQR)^c^	118 (100-133)	121 (107-134)	108 (89-128)	.002	11 (4 to 19)	—

^a^Not applicable.

^b^QoR-15: quality of recovery-15.

^c^Data available: total, n=85; low-risk patients, n=59; high-risk patients, n=26. This indicates the number included in the analysis.

### Risk Stratification Performance

In terms of performance, with an incidence of opioid use of >90 MME/day as the primary outcome, the pilot risk stratification algorithm was reasonably specific (true negative rate=144/200, 72%) but not sensitive (true positive rate=10/31, 32%). These equate to a high false negative rate of 68% (21/31), with a false positive rate of 28% (56/200), a positive likelihood of 1.15, and a negative likelihood of 0.94.

### Postoperative Use of the POQI Platform

Data are available for 214 (77.5%) of the 276 patients who completed the HHQ and were risk stratified by the POQI platform (low-risk patients: 161/203, 79.3%; high-risk patients: 53/73, 73%). Of the 276 patients, 85 (30.8%) completed any postoperative QoR-15 questionnaire (low-risk patients: 59/203, 29.1%; high-risk patients: 26/73, 36%). Similarly, 31 (15.3%) of the 203 low-risk patients and 3 (4.1%) of the 73 high-risk patients reported any postoperative opioid use (Table 6).

**Table 6:** Postoperative use of the perioperative quality improvement (POQI) platform.

	Total sample (n=276)	Low-risk group (n=203)	High-risk group (n=73)	*P* value	Median difference (95% CI)	Odds ratio (95% CI)
Data available from preoperative HHQ^a^, n (%)	214 (77.5)	161 (79.3)	53 (72.6)	.26	—^b^	0.7 (0.4 to 1.4)
Completed at least 1 postoperative questionnaire, n (%)	85 (30.8)	59 (29.1)	26 (35.6)	.62	—	0.9 (0.4 to 1.7)
Length of follow-up postsurgery (days), median (IQR)^c^	25 (11-54)	24 (11-53)	29 (11-57)	.80	–1 (–9 to 10)	—
Completed follow-up questionnaires at POD^d^ 31 to 60, n (%)	15 (5.4)	11 (5.4)	4 (5.5)	.99	—	1.0 (0.3 to 4.4)
Completed follow-up questionnaires beyond POD 90, n (%)	3 (1.1)	0 (0)	3 (4.1)	.57	—	0 (0 to 6.8)
Patients reporting postoperative medication use, n (%)	34 (12.3)	31 (15.3)	3 (4.1)	.01	—	4.2 (1.2 to 22.1)

^a^HHQ: health history questionnaire.

^b^Not applicable.

^c^Data available: total, n=85; low-risk patients, n=59; high-risk patients, n=26. This indicates the number included in the analysis.

^d^POD: postoperative day.

### Qualitative Interviews

We conducted feedback interviews with 3 (15%) patients (2 POQI-L users and 1 POQI-H user) of the 20 invited patients; most patients (17/20, 85%) approached declined to participate in this portion of the study. We interviewed all 4 clinicians (anesthesiologists and nurses who used both platforms) involved in the platform deployment in St. Paul’s Hospital Transitional Pain Clinic.

### Perceived Benefit of the Platforms for the Health Care Experience

Patients recognized that the POQI-L had improved their health care experience by making them mindful of their behavior, such as “stating how I was feeling, anxiety about things, etc*,”* which gave them “a sense of agency” over their care. It also provided a sense of reassurance that the health care team was continually monitoring their health status after they returned home following hospital discharge. Similarly, the POQI-H user believed there was a potential benefit:

[T]his will help me keep track of things and have some kind of two-way communication

However, they did not feel that the potential had been met with the current version.

The clinical users perceived minimal benefits of the POQI-H, such as improving their workflows and allowing them to manage their patients better. However, they recognized potential patient benefits, including access to educational information:

[F]or the patients, there is good access to many resources.

[The platform] provided people with resources to manage their pain well while they’re at home [with] an option to access further information

as needed

The clinical users identified benefits of the POQI-L, which administered the HHQ to all patients as a screening and triage tool: clinicians reported that it was helpful to display the pain risk score and “to see whether they’re a high or low risk as a quick way to screen patients*.*” Integrating patient information in a single document was also helpful:

[It was] also useful as a way to gather all the patient’s medical history.

### User Experience With the Platforms

Patients experienced issues using both platforms, although this may have resulted from poor communication of the purpose of the application and potential benefits for them:

I’m not sure what that tool is trying to be.

POQI-L user

[...] I didn’t feel like I had much guidance in using [it].

POQI-H user

Furthermore, there was a lack of clarity in instructions for using both platforms; for example, the POQI-L users expressed frustration about redundant emails or SMS text messages, which were unclear about “what was supposed to be completed and when*,*” and the POQI-H user said as follows:

I wasn’t sure if I was supposed to initiate certain things, or if like somebody from my care team would go in.

Furthermore, the 2 POQI-L participants were unaware of their postoperative risk score and its details and viewed this as a missed opportunity to benefit from understanding their personalized risk for significant postsurgical pain.

Similarly, usability issues during the initial deployment contributed to attrition among clinical users; for example, 1 clinician admitted that they had not signed patients up on the POQI-H for 4 months, as they did not find it easy to use, were not satisfied with the functionality, and could not quickly locate necessary information; another clinician had “*stopped using [POQI-L] as a method to look up patients and filter them out to see who should be put on [POQI-H].*” The clinicians who had used both platforms expressed concerns with quality assurance and usability:

*I think both platforms have much potential when they’re working*... *[but]there have been many [issues] to deal with in the development of the programs, which have been both challenging and frustrating*.

Both patients and clinicians expressed a desire for greater platform integration. One patient stated as follows:

[I] would have hoped that there would have been things populated in it [to] show the integration of services that I was accessing post-surgery.

Clinicians indicated that there should be a single platform with a unified vision; for example, a clinician stated as follows:

I want to be able to do everything from one platform; I don’t want to have to be on multiple different platforms. So that’s my ideal scenario.

## Discussion

### Principal Findings

A pragmatic risk prediction algorithm was used to categorize 276 patients who underwent colorectal surgery into high-risk or low-risk groups for significant postoperative pain. The algorithm’s performance was evaluated using a primary outcome threshold of >90 MME/day during in-hospital recovery: it was found to be reasonably specific (true negative rate=14/200, 72%) but not sensitive (true positive rate=10/31, 32%). Furthermore, the risk categorization was used to drive dedicated preoperative and postoperative patient surveys using the high-risk (POQI-H) or low-risk (POQI-L) platforms. Preoperative surveys, including HHQ, were completed by 214 (77.5%) of the 276 patients, but there was a significant loss to follow-up with postoperative surveys, including QoR-15, completed by only 85 (39.7%) of the 214 patients. Qualitative feedback from clinician and patient users indicated shortcomings in the design and implementation of the patient- and clinician-facing components of the POQI platform.

### Comparison With Prior Work

The motivation was that POQI would establish a platform to support personalized multimodal pain management techniques and patient preparation or education to reduce reliance on opioids (both in-hospital and postdischarge opioid use) during recovery from surgery. Identifying those at most significant risk of postoperative pain and providing tailored care plans based on their risk levels may help reduce initial opioid consumption. A recent systematic review suggested that a higher risk of developing persistent postsurgical pain is associated with younger age, female sex, and preoperative pain [25], which are consistent with the characteristics observed in the patients classified as high risk by our algorithm (Table 2). Furthermore, a recent multicenter study in the United States identified preoperative opioid use as the most significant predictor of prolonged opioid use after surgery [26]. Again, this factor was a significant distinguishing characteristic of our high-risk patients, along with a history of depression, antidepressant use, and chronic pain (Table 2).

Virtual care solutions for patients in the postsurgical period, including web-based tools and mobile apps, can support tracking various postoperative outcomes, including prescription drug use. Although the development of perioperative eHealth or mobile health solutions for telemonitoring is still maturing [27], these technologies show promise as not only their implementation is feasible but they can also streamline clinical workflow and improve patient outcomes [28,29]. Web-based patient portals integrated with the EMR can improve patient satisfaction, enable more effective health care use [30], and improve outcomes such as glycemic control in patients with diabetes [31]. However, there are several barriers to successful implementation, as our experience with poor patient retention indicates (Figure 3). To improve patient engagement through an EMR portal, it is essential to avoid high attrition rates, which requires addressing the requirements of diverse patients, focusing on usability and functionality, and adopting implementation science approaches [32]; using apps can also have a positive impact [33]. Perioperative solutions must be designed with frequent and meaningful clinician and patient input and evaluated in large, robust clinical trials [27,29]. Particular attention is needed when developing and evaluating tools for vulnerable populations, such as patients with chronic pain issues and older patients, although a recent systematic review reported generally positive results from 7 studies on patients aged ≥65 years [34]. In contrast, our population was relatively younger, with a median age of 59 (IQR) (47-68) years. Furthermore, an evaluation of a patient-centric digital pain management app reported acceptable patient engagement and improved anxiety and pain catastrophizing in similarly aged patients who had experienced chronic pain of moderate to severe intensity for at least 3 months [33].

The lack of follow-up data prevented us from effectively evaluating or optimizing the risk stratification algorithm we implemented. The risk model was reasonably specific, based on in-hospital MME, but with poor sensitivity and a subsequent high false negative rate, as it failed to identify patients who may have benefited from the POQI-H platform. None of our 11 patient-reported preoperative risk factors had a significant adjusted OR for high in-hospital opioid use (>90 MME/day), in which the 95% CI range excluded 1 (Table 4). This indicates that by themselves, none of the risk factors would have predicted high postoperative opioid use in this cohort, although these are recognized risk factors. This contradicts the findings from our retrospective study in the same hospital, which found that a history of chronic pain and substance use disorder was associated with high postoperative opioid requirements [20]. The small sample sizes in both our retrospective and prospective cohorts may have limited our ability to detect these associations reliably in the chosen population. Alternatively, despite being evidence based [24], our selected threshold of >90 MME/day may not be optimal. Future work should explore other potentially self-reportable risk factors, such as open surgery, pain catastrophizing, or lack of planned regional anesthesia, as well as interactions between synergistic or antagonistic risk factors. Finally, data science approaches show promise in predicting postsurgical outcomes, with generally positive findings in a recent systematic review [35]. Such technology has been used to predict prolonged opioid use after orthopedic surgery [36] or estimate the risk of an adverse outcome within 30 days of an opioid dispensation [37]. These techniques may help refine local models, such as our algorithm, but we need more data at this stage.

Importantly, our platform was an amalgamation of various existing (or slightly adapted) technologies that lacked adequate workflow integration and did not adapt to varying clinical or patient needs to allow evaluation when there were any deviations from the predefined workflow. For example, we could not access clinically relevant long-term outcomes for many high-risk patients. Improving access to available administrative and clinical data could facilitate improved prediction performance using machine learning techniques [37].

### Lessons Learned

We cannot report a fully realized solution due to a lack of integration with the provincial medication system and the reduced scope of the platform in light of the COVID-19 pandemic. However, the problems that we encountered and the lessons learned during our implementation can benefit other research, specifically clinical and industry teams endeavoring to build perioperative virtual care solutions to improve postoperative opioid use after discharge. Any work addressing this critical public health problem should ensure frequent engagement of patient and clinical partners, including co-design [38], to confirm that the design addresses patient and provider needs and delivers meaningful benefits to patient care and health care practice.

Next, when including a research component in health care system technical development and implementation, it is essential to ensure that research end points are integrated into project plans. This ensures that industry partners and clinical teams contribute to and approve evaluation plans so that the teams understand and support each other’s priorities. We also suggest including all partners in frequent data quality assessments and using an objective committee to oversee project activities, focusing on system-level goals while enabling each partner to achieve their respective objectives.

Given the likelihood that the requirement for virtual care solutions in the perioperative setting will grow, preparing for the transition to a long-term sustainable implementation is essential [39,40]. This should leverage experiences from stakeholders; focus on user experience; and ensure data are collected, validated, and delivered to the right people at the right time to improve the quality of care. Feedback is essential to a learning health system [41]: process metrics, patient trajectories, and benchmarking tools will enable clinicians to learn from their patients. PROMs and patient-reported experience measures [42] will be fundamental to improving the quality of care provided, focusing on patient-relevant outcomes rather than only system-relevant ones and enabling the personalization of care.

### Limitations

In addition to the implementation issues already discussed, we must acknowledge many limitations in the data that we have presented. First, restrictions to hospital access due to the COVID-19 pandemic care considerations leading up to and during the pilot recruitment period likely caused significant delays. It also hampered effective engagement between patients, the research team, clinical teams, and industry partners and disrupted the opportunity to refine the software solution through further design iterations.

Second, it is unclear from our data how patients used the information provided through the platform. The qualitative results from a limited number of patients willing to be interviewed and clinicians suggest that some patients glimpsed the potential value of the tool. However, they did not use or benefit from the educational materials and saw the platform as a survey tool rather than a virtual care platform. This may have contributed to the observed attrition rate and lack of interest in participating in usability interviews. Further design iterations were needed to respond to end user concerns and improve engagement in the platform. The lack of long-term follow-up was further compounded by technical issues and the lack of completed PROM survey data from patients. To prevent this from happening in the future, it may be better to engage and support patients’ needs through a prospective approach that uses a near real-time data pipeline and integrated interfaces directly into workflows at the point of care. The lack of bidirectional EMR integration is a limitation of our implementation. It likely contributed to our high attrition rates and compromised the quality of the data we could report on. As discussed, improving patient engagement through an EMR portal requires a more robust implementation approach than we could apply here.

Third, the primary aim of the algorithm to identify persistent postoperative opioid requirements could not be determined without access to prescription data to verify dispensed medications after discharge. Gaining such access using patient-directed or authorized access through the British Columbia Health Gateway was a project goal, and implementation was explored. However, it was found to be impossible due to provincial policy constraints. Hence, we cannot know whether the intervention impacted prolonged opioid use after surgery. Future studies should explicitly include long-term follow-up but may have to augment it with self-reports to capture the difference between dispensed and taken medications.

Finally, this analysis is limited due to a small sample size from a single center (including only 24, 11.9% of the 201 patients who used opioids >90 MME/day) and missing follow-up outcomes from many patients designated as high risk for significant postsurgical pain and opioid use. This is partly due to low engagement during the COVID-19 pandemic and challenges in achieving the project’s objectives within a limited funding period. Similarly, we planned to recruit 10 patients from the POQI-L group, 10 from the POQI-H group, and 5 clinicians to participate in semistructured interviews. However, we only obtained feedback from 3 patients (2 POQI-L users and 1 POQI-H user) and 4 clinicians. A broader sample would have provided more insight into the shortcomings and potential benefits of the system and should be built into any future evaluation.

Again, this final limitation was, at least in part, due to the COVID-19 pandemic. On the other hand, the COVID-19 pandemic created a greater motivation for developing and implementing systems that support virtual care through the perioperative process. This may be particularly relevant in a hospital such as St. Paul’s Hospital, a tertiary care academic hospital with patients from all over British Columbia, a geographically vast Canadian province with a widely distributed population. Finally, pain management requires multidisciplinary care that may not be available in rural communities. A well-designed platform could fill this gap and enable patients to benefit from personalized risk prediction and virtual prehabilitation while overcoming potential resource constraints.

### Conclusions

Our POQI platform categorized patients who underwent colorectal surgery into high-risk or low-risk groups for significant postoperative pain and opioid use, using a pragmatic risk prediction algorithm. The algorithm’s performance was reasonably specific but not sensitive in predicting in-hospital opioid requirements. However, a significant loss in follow-up with postdischarge surveys suggested shortcomings in the design and implementation of the platform, which may have been improved with additional development work and the opportunity to engage patients more comprehensively. Important lessons learned during implementation included the early and frequent engagement of patients and clinical partners in the design and evaluation process. Finally, POQI platform users appreciated its potential impact on reducing opioid exposure, streamlining perioperative care, and improving patient outcomes, suggesting a redesign and evaluation before wider implementation is desirable.

## Appendix

Multimedia Appendix 1 Risk factors and relevant questions from health history questionnaire.

Multimedia Appendix 2 List of included colorectal surgeries in pilot implementation.

Multimedia Appendix 3 Morphine milligram equivalent conversion factors.

Multimedia Appendix 4 Interview guides.

## Abbreviations

EMR: electronic medical record
ERAS: enhanced recovery after surgery
HHQ: health history questionnaire
MD: median difference
MME: morphine milligram equivalents
OR: odds ratio
PHC: Providence Health Care
POQI: Perioperative Opioid Quality Improvement
POQI-H: Perioperative Opioid Quality Improvement platform for high-risk patients
POQI-L: Perioperative Opioid Quality Improvement platform for low-risk patients
PROM: patient-reported outcome measure
QoR-15: quality of recovery-15
SQUIRE: Standards for Quality Improvement Reporting Excellence

## References

[1] (2021). Drug overdose deaths in the U.S. top 100,000 annually. CDC/National Center for Health Statistics.

[2] (2024). Opioid- and stimulant-related harms in Canada. Public Health Agency of Canada.

[3] Soffin EM, Lee BH, Kumar KK, Wu CL (2019). The prescription opioid crisis: role of the anaesthesiologist in reducing opioid use and misuse. Br J Anaesth.

[4] Elhassan A, Elhassan I, Elhassan A, Sekar KD, Cornett EM, Urman RD, Kaye AD (2019). Perioperative surgical home models and enhanced recovery after surgery. J Anaesthesiol Clin Pharmacol.

[5] Beverly A, Kaye AD, Ljungqvist O, Urman RD (2017). Essential elements of multimodal analgesia in enhanced recovery after surgery (ERAS) guidelines. Anesthesiol Clin.

[6] King AB, Alvis BD, McEvoy MD (2016). Enhanced recovery after surgery, perioperative medicine, and the perioperative surgical home: current state and future implications for education and training. Curr Opin Anaesthesiol.

[7] Huang CC, Sun WZ, Wong CS (2018). Prevention of chronic postsurgical pain: the effect of preventive and multimodal analgesia. Asian J Anesthesiol.

[8] Pozek JP, Beausang D, Baratta JL, Viscusi ER (2016). The acute to chronic pain transition: can chronic pain be prevented?. Med Clin North Am.

[9] Curatolo M (2016). Regional anesthesia in pain management. Curr Opin Anaesthesiol.

[10] Moorthy K, Wynter-Blyth V (2017). Prehabilitation in perioperative care. Br J Surg.

[11] Minnella EM, Carli F (2018). Prehabilitation and functional recovery for colorectal cancer patients. Eur J Surg Oncol.

[12] Wynter-Blyth V, Moorthy K (2017). Prehabilitation: preparing patients for surgery. BMJ.

[13] Hah JM, Bateman BT, Ratliff J, Curtin C, Sun E (2017). Chronic opioid use after surgery: implications for perioperative management in the face of the opioid epidemic. Anesth Analg.

[14] Stark N, Kerr S, Stevens J (2017). Prevalence and predictors of persistent post-surgical opioid use: a prospective observational cohort study. Anaesth Intensive Care.

[15] Sun EC, Darnall BD, Baker LC, Mackey S (2016). Incidence of and risk factors for chronic opioid use among opioid-naive patients in the postoperative period. JAMA Intern Med.

[16] Macrae WA (2001). Chronic pain after surgery. Br J Anaesth.

[17] Kehlet H, Jensen TS, Woolf CJ (2006). Persistent postsurgical pain: risk factors and prevention. Lancet.

[18] Yefet LS, Bone JN, Courtemanche R, Lauder G, Courtemanche DJ (2021). Opioid prescribing patterns in British Columbia from 2013 to 2017: a population-based study. B C Med J.

[19] Edwards NY, Sutherland AM, Caters L, Kim LS, Chan S, Shetty S, Flexman AM, Kim J (2023). Opioid overdose following surgery or pain treatment: a missed opportunity for intervention. BC Med J.

[20] Sreepada R, Sutherland A, Shams B, Soriano G, Perrett E, Chowdhury N, Wood M, Kim J, Görges M (2021). Development of a simple risk prediction model for excessive postoperative opioid utilization in inpatients. Anesth Analg.

[21] TCPS 2 (2022) – chapter 2: scope and approach. The Panel on Research Ethics, Canada.

[22] Ogrinc G, Davies L, Goodman D, Batalden P, Davidoff F, Stevens D (2016). SQUIRE 2.0 (standards for QUality Improvement reporting excellence): revised publication guidelines from a detailed consensus process. BMJ Qual Saf.

[23] Stark PA, Myles PS, Burke JA (2013). Development and psychometric evaluation of a postoperative quality of recovery score: the QoR-15. Anesthesiology.

[24] Busse JW, Craigie S, Juurlink DN, Buckley DN, Wang L, Couban RJ, Agoritsas T, Akl EA, Carrasco-Labra A, Cooper L, Cull C, da Costa BR, Frank JW, Grant G, Iorio A, Persaud N, Stern S, Tugwell P, Vandvik PO, Guyatt GH (2017). Guideline for opioid therapy and chronic noncancer pain. CMAJ.

[25] Andreoletti H, Dereu D, Combescure C, Rehberg B (2022). A systematic review and meta-analysis of three risk factors for chronic postsurgical pain: age, sex and preoperative pain. Minerva Anestesiol.

[26] Kuck K, Naik BI, Domino KB, Posner KL, Saager L, Stuart AR, Johnson KB, Alpert SB, Durieux ME, Sinha AK, Brummett CM, Aziz MF, Cummings KC, Gaudet JG, Kurz A, Rijsdijk M, Wanderer JP, Pace NL, Multicenter Perioperative Outcomes Group Enhanced Observation Study Investigator Group for the Multicenter Perioperative Outcomes Group Enhanced Observation Study Collaborator Group (2023). Prolonged opioid use and pain outcome and associated factors after surgery under general anesthesia: a prospective cohort association multicenter study. Anesthesiology.

[27] Haveman ME, Jonker LT, Hermens HJ, Tabak M, de Vries JP (2024). Effectiveness of current perioperative telemonitoring on postoperative outcome in patients undergoing major abdominal surgery: a systematic review of controlled trials. J Telemed Telecare.

[28] van der Meij E, Anema JR, Otten RH, Huirne JA, Schaafsma FG (2016). The effect of perioperative E-Health interventions on the postoperative course: a systematic review of randomised and non-randomised controlled trials. PLoS One.

[29] De La Cruz Monroy MF, Mosahebi A (2019). The use of smartphone applications (Apps) for enhancing communication with surgical patients: a systematic review of the literature. Surg Innov.

[30] Graham TA, Ali S, Avdagovska M, Ballermann M (2020). Effects of a web-based patient portal on patient satisfaction and missed appointment rates: survey study. J Med Internet Res.

[31] Alturkistani A, Qavi A, Anyanwu PE, Greenfield G, Greaves F, Costelloe C (2020). Patient portal functionalities and patient outcomes among patients with diabetes: systematic review. J Med Internet Res.

[32] Lyles CR, Nelson EC, Frampton S, Dykes PC, Cemballi AG, Sarkar U (2020). Using electronic health record portals to improve patient engagement: research priorities and best practices. Ann Intern Med.

[33] Bhatia A, Kara J, Janmohamed T, Prabhu A, Lebovic G, Katz J, Clarke H (2021). User engagement and clinical impact of the manage my pain app in patients with chronic pain: a real-world, multi-site trial. JMIR Mhealth Uhealth.

[34] Jonker LT, Haveman ME, de Bock GH, van Leeuwen BL, Lahr MM (2020). Feasibility of perioperative eHealth interventions for older surgical patients: a systematic review. J Am Med Dir Assoc.

[35] Elfanagely O, Toyoda Y, Othman S, Mellia JA, Basta M, Liu T, Kording K, Ungar L, Fischer JP (2021). Machine learning and surgical outcomes prediction: a systematic review. J Surg Res.

[36] Anderson AB, Grazal CF, Balazs GC, Potter BK, Dickens JF, Forsberg JA (2020). Can Predictive Modeling Tools Identify Patients at High Risk of Prolonged Opioid Use After ACL Reconstruction?. Clin Orthop Relat Res.

[37] Sharma V, Kulkarni V, Jess E, Gilani F, Eurich D, Simpson SH, Voaklander D, Semenchuk M, London C, Samanani S (2022). Development and validation of a machine learning model to estimate risk of adverse outcomes within 30 days of opioid dispensation. JAMA Netw Open.

[38] Knowles SE, Allen D, Donnelly A, Flynn J, Gallacher K, Lewis A, McCorkle G, Mistry M, Walkington P, Brunton L (2022). Participatory codesign of patient involvement in a learning health system: how can data-driven care be patient-driven care?. Health Expect.

[39] Shaw J, Brewer LC, Veinot T (2021). Recommendations for health equity and virtual care arising from the COVID-19 pandemic: narrative review. JMIR Form Res.

[40] Dorn SD (2021). Backslide or forward progress? Virtual care at U.S. healthcare systems beyond the COVID-19 pandemic. NPJ Digit Med.

[41] Enticott J, Johnson A, Teede H (2021). Learning health systems using data to drive healthcare improvement and impact: a systematic review. BMC Health Serv Res.

[42] Kingsley C, Patel S (2017). Patient-reported outcome measures and patient-reported experience measures. BJA Educ.

