# Supplementary material for: Postsurgical Pain Risk Stratification to Enhance Pain Management Workflow in Adult Patients: Design, Implementation, and Pilot Evaluation
Source: JMIR Perioper Med. 2024 Jul 2;7:e54926. doi: 10.2196/54926 (PMC11252618; doi:10.2196/54926)
Supplement: Multimedia Appendix 1 [file periop_v7i1e54926_app1.docx]

# Multimedia Appendix 1: Risk Factors and relevant question from Health History Questionnaire

The conditions necessary to meet the score are bolded in the details below.

## Risk factor: Current prescription of opioid (weight=5)

My surgeries

3. Pain Screening

The following questions are part of a screening tool to help understand your pain risk before and after surgery.

- Are you currently taking any opioids? (Please click "Yes" if you are unsure and review the options) (**yes**/no)
- *If yes:* Which of these opioids are you currently taking? (?)
  - Dilaudid (Hydromorphone)
  - Hydromorphone Contin
  - Percocet (Oxycodone & Acetaminophen)
  - Suprudol (oxycodone)
  - Oxycontin
  - Statex (morphine)
  - M-Eslon/Kadian (morphine extended-release)
  - Tramacet (tramadol & acetaminophen)
  - Durela/Tridural (tramadol)
  - Emtec/Tylenol 3 (codeine & acetaminophen)
  - Duragesic (fentanyl)

## Risk factor: Benzodiazepine prescription (weight=4)

My surgeries

3. Pain Screening

The following questions are part of a screening tool to help understand your pain risk before and after surgery.

- Are you currently taking any benzodiazepines (Please click "Yes" if you are unsure and review the options) (?) (**yes**/no)
- *If yes:* Which benzodiazepines are you currently taking?
  - Ativan (Lorazepam)
  - Clonazepam
  - Alprazolam
  - Valium (Diazepam)
  - Oxazepam

## Risk factor: Antidepressant prescription (weight=4);

My surgeries

3. Pain Screening

The following questions are part of a screening tool to help understand your pain risk before and after surgery.

- Are you currently taking any depressants? (**yes**/no)
- *If yes:* Which antidepressants are you currently taking?
  - Citalopram
  - Cipralex (Escitalopram)
  - Prozac (Fluoxetine)
  - Luvox (Fluvoxamine)
  - Paxil (Paroxetine)
  - Zoloft (Sertraline)
  - Elavil (Amitriptyline)
  - Aventyl (Nortriptyline)
  - Pristiq (Desvenlafaxine)
  - Cymbalta (Fluoxetine)
  - Effexor (Venlafaxine)
  - Wellbutrin (Bupropion)

## Risk factor: History of chronic pain (weight=4)

My surgeries

3. Pain Screening

The following questions are part of a screening tool to help understand your pain risk before and after surgery.

- Do you have or have ever had:
  - Chronic pain? (**yes**/no)

**OR**

My muscles, Joints, and Nerves

Click Yes, if you have, or have ever had, any of these conditions:

- Chronic pain (**yes**/no)

**OR**

- Do you suffer from chronic pain? (**yes**/no)

## Risk factor: Anxiety or panic attacks (weight=2)

My Well-being

- Do you suffer from anxiety or panic attacks? (**yes**/no)

**OR**

My Well-being

- Do you have any mental health conditions? (**yes**/no)
- *If yes:* Please identify your mental health conditions.
  - Depression
  - **Generalized Anxiety Disorder**
  - **Panic Attacks**
  - Bipolar Disorder
  - Schizophrenia
  - Obsessive-Compulsive Disorder
  - Dementia
  - Alzheimer’s
  - Previous Psychosis
  - Mood Disorder
  - Add Other

## Risk factor: History of depression (weight=2)

My surgeries

3. Pain Screening

The following questions are part of a screening tool to help understand your pain risk before and after surgery.

- Do you have or have ever had:
- Depression? (**yes**/no)

**OR**

My Well-being

- Do you have any mental health conditions? (**yes**/no)
- *If yes:* Please identify your mental health conditions.
  - **Depression**
  - Generalized Anxiety Disorder
  - Panic Attacks
  - Bipolar Disorder
  - Schizophrenia
  - Obsessive-Compulsive Disorder
  - Dementia
  - Alzheimer’s
  - Previous Psychosis
  - Mood Disorder
  - Add Other

## Risk factor: Poorly controlled pain after surgery (weight=2)

My surgeries

3. Pain Screening

The following questions are part of a screening tool to help understand your pain risk before and after surgery.

- Have you ever had pain that was hard to control after surgery? (**yes**/no)

## Risk factor: Open surgery (weight=0)

My surgeries

3. Pain Screening

The following questions are part of a screening tool to help understand your pain risk before and after surgery.

- Are you having an open surgery? (?) (**yes**/no)

## Risk factor: Age <40 years (weight=1)

Uses account registration information (calculated from date of birth)

- Age - Patient's age (**value < 40**)

## Risk factor: Female sex (weight=2);

Uses account registration information

- Sex assigned at birth (**female**)

## Risk factor: Substance use disorder (weight=5);

My surgeries

3. Pain Screening

The following questions are part of a screening tool to help understand your pain risk before and after surgery.

- Substance use disorder? (?) (**yes**/no)

## Risk factor: Recreational drug use (weight=4);

My Lifestyle

- Do you use any recreational drugs? (**yes**/no)
- *If yes:* Please identify which substances you use
  - Heroin
  - Fentanyl
  - Amphetamines
  - Cocaine
  - Marijuana
  - Any narcotics
  - MDMA
  - IV/injectable recreational drugs
  - Add other
