# Supplementary material for: Postsurgical Pain Risk Stratification to Enhance Pain Management Workflow in Adult Patients: Design, Implementation, and Pilot Evaluation
Source: JMIR Perioper Med. 2024 Jul 2;7:e54926. doi: 10.2196/54926 (PMC11252618; doi:10.2196/54926)
Supplement: Multimedia Appendix 2 [file periop_v7i1e54926_app2.docx]

# Multimedia Appendix 2: List of included colorectal surgeries in the pilot implementation

| Adrenalectomy | Fasciotomy Lower Extremity Bilateral | Proctocolectomy Laparoscopic | Repair Rectal Prolapse Rectal Approach |
| --- | --- | --- | --- |
| Adrenalectomy Retroperitoneal Laparoscopic | Fasciotomy Upper Extremity | Proctopexy | Resection Abdominoperineal |
| Anoplasty | Fistulectomy Anus | Pullthrough Endorectal | Resection Abdominoperineal Laparoscopic |
| Anorectoplasty Pena Posterior Sagittal | Fundoplication Nissen | Pullthrough Endorectal Laparoscopic | Resection Abdominoperineal Laparoscopic with Transanal Microsurgery Assisted |
| Anorectoplasty Pena Posterior Sagittal Laparoscopic | Fundoplication Nissen Laparoscopic | Pyloromyotomy | Resection Anterior Colon Laparoscopic |
| Appendectomy | Gastrectomy | Pyloromyotomy Laproscopic | Resection Bowel Abdomino Perineal (Abdominal Portion) |
| Appendectomy Laparoscopic | Gastrectomy Laparoscopic | Pyloroplasty | Resection Bowel Abdomino Perineal (Perineal Portion) |
| Bishop Koop Procedure | Gastrectomy Partial | Reanastomosis Hartmann Lithotomy | Resection Bowel Laparoscopic Lithotomy |
| Cholecystectomy | Gastrectomy Partial Laparoscopic | Reanastomosis Hartmann Supine | Resection Bowel Laparoscopic Supine |
| Cholecystectomy Laparoscopic | Gastrojejunostomy | Reconstruction Esophagus | Resection Colectomy Laparoscopic |
| Cholecystectomy with Cholangiogram | Gastrostomy | Removal Gastric Band Laparoscopic | Resection Colon Subtotal |
| Cholecystectomy with Cholangiogram Laparoscopic | Gastrostomy Laparoscopic | Removal Pectus Excavatum Nuss Bar | Resection Intragastric Tumor Laparoscopic and Endoscopic |
| Choledochojejunostomy | Gastrostomy Percutaneous Endoscopic | Repair Atresia Esophagus | Resection Large Bowel |
| Choledochojejunostomy Laparoscopic | Hartmann Procedure Laparoscopic | Repair Duodenal Ulcer | Resection Large Bowel Laparoscopic |
| Closure Bishop Koop Laparoscopic | Hemicolectomy Left | Repair Fistula Rectovaginal with Graciloplasty | Resection Lesion Rectum Transanal Assisted Microsurgery |
| Closure Colostomy | Hemicolectomy Right | Repair Gastric Ulcer | Resection Liver Segmental |
| Closure Colostomy Laparoscopic | Hemorrhoidectomy | Repair Hernia Diaphragm | Resection Low Anterior Colon |
| Closure Fistula Gastrocutaneous | Hepatoportoenterostomy | Repair Hernia Epigastric | Resection Low Anterior Colon Lithotomy |
| Closure Gastroschisis | Ileostomy | Repair Hernia Femoral Bilateral | Resection Low Anterior Transanal Assisted Laparascopic |
| Closure Ileostomy | Ileostomy Laparoscopic | Repair Hernia Hiatal | Resection Retroperitoneal Mass |
| Closure Ileostomy Laparoscopic | Incision and Drainage Lower Extremity | Repair Hernia Inguinal | Resection Retroperitoneal Mass Laparoscopic |
| Closure Jejunostomy | Incision and Drainage Perianal Fistula | Repair Hernia Inguinal Bilateral | Resection Sigmoid Colon |
| Closure Jejunostomy Laparoscopic | Incision and Drainage Perineum | Repair Hernia Inguinal Bilateral Laparoscopic | Resection Sigmoid Hartmann |
| Closure Loop Ileostomy | Incision and Drainage Rectal Abscess | Repair Hernia Inguinal Laparoscopic | Resection Small Bowel |
| Colostomy | Incision and Drainage Upper Extremity | Repair Hernia Parasternal | Resection Small Bowel Laparoscopic |
| Colostomy Laparoscopic | Insertion Jejunostomy Tube Laparoscopic | Repair Hernia Parasternal Laparoscopic | Resection Total Colectomy |
| Creation Pelvic Pouch | Ladds Procedure | Repair Hernia Parastomal | Reversal Hartmann Procedure Laparoscopic |
| Creation Pelvic Pouch Laparoscopic | Laparoscopy | Repair Hernia Parastomal Laparoscopic | Revision Colostomy |
| Dissection Lymph Node Inguinal | Laparoscopy Diagnostic | Repair Hernia Umbilical | Revision Stoma |
| Emergency Resection Bowel | Laparotomy Exploratory | Repair Hernia Ventral | Roux-En-Y Laparoscopic |
| Enteroenterostomy | Lysis Adhesions | Repair Hernia Ventral Laparoscopic | Sphincterectomy |
| Esophagogastrectomy | Lysis Adhesions Abdomen Laparoscopic | Repair Omphalocele | Sphincteroplasty Anal |
| Excision Adenoma Transanal | Myotomy Heller Laparoscopic | Repair Perforated Ulcer Laparoscopic | Sphincterotomy |
| Excision Cyst Choledochal | Omentectomy | Repair Perineal Defect with Flap | Vagotomy with Pyloroplasty |
| Excision Cyst Duplication Laparoscopic | Pancreatectomy | Repair Rectal Prolapse Abdominal Approach |  |
| Excision Transanal Laparoscopic | Pancreaticoduodenectomy | Repair Rectal Prolapse Abdominal Approach Laparoscopic |  |
| Exenteration Pelvic Cavity | Proctocolectomy | Repair Rectal Prolapse Perineal Approach Laparoscopic |  |
