# Supplementary material for: Postsurgical Pain Risk Stratification to Enhance Pain Management Workflow in Adult Patients: Design, Implementation, and Pilot Evaluation
Source: JMIR Perioper Med. 2024 Jul 2;7:e54926. doi: 10.2196/54926 (PMC11252618; doi:10.2196/54926)
Supplement: Multimedia Appendix 3 [file periop_v7i1e54926_app3.docx]

# Multimedia Appendix 3: Morphine Milligram Equivalent (MME) Conversion Factors

## Opioid Oral MME Conversion Factor

| **Opioids** | **Unit** | **Conversion Factor** |
| --- | --- | --- |
| oxycodone | mg | 1.5 |
| morphine | mg | 1 |
| methadone | Below 21 mg | 4 |
| methadone | Between 21-40 mg | 8 |
| methadone | Between 41-60 mg | 10 |
| methadone | Over 60 mg | 12 |
| meperidine | mg | 0.1 |
| hydromorphone | mg | 5 |
| fentanyl | mcg | 0.1 |
| codeine | mg | 0.15 |

## Opioid IV MME Conversion Factor

[Health Canada](https://recalls-rappels.canada.ca/en/alert-recall/fentanyl-transdermal-systems-new-changes-dose-conversion-guidelines-health-care)* recommends a conversion ratio of 1:3 for oral/subcutaneous to IV opioid administration.

| **Opioids** | **Unit** | **Conversion Factor** |
| --- | --- | --- |
| morphine | mg | 3 |
| hydromorphone | mg | 15 |
| fentanyl (Below 21 mg) | mg | 0.3 |

* <https://recalls-rappels.canada.ca/en/alert-recall/fentanyl-transdermal-systems-new-changes-dose-conversion-guidelines-health-care>; specifically, <https://recalls-rappels.canada.ca/en/alert-recall/fentanyl-transdermal-systems-new-changes-dose-conversion-guidelines-health-care#fn_t1b1-ref>. See also <https://recalls-rappels.canada.ca/en/alert-recall/important-changes-dose-conversion-guidelines-fentanyl-transdermal-systems-health#conversion>
