# Supplementary material for: Postsurgical Pain Risk Stratification to Enhance Pain Management Workflow in Adult Patients: Design, Implementation, and Pilot Evaluation
Source: JMIR Perioper Med. 2024 Jul 2;7:e54926. doi: 10.2196/54926 (PMC11252618; doi:10.2196/54926)
Supplement: Multimedia Appendix 4 [file periop_v7i1e54926_app4.docx]

# Multimedia Appendix 4: Interview guides

##

## Interview Guide (Patient version)

Tasks for each telephone interview:

1. Ask to speak with the consented patient and have the speaker confirm.
2. Confirm now is a convenient and safe time to talk and that they consent to speak with you for approximately 10-15 minutes; otherwise re-arrange for a later date.
3. Explain the format of the brief interview (over Zoom or telephone) and that you will be using Zoom’s live transcription function or taking handwritten notes to record their answers and will ask additional clarifying questions as needed.
4. Thank them for their participation and their willingness to speak today.

## Participant introduction for each session:

“Good morning/afternoon/evening, thank you for taking the time to join us today. My name is <your name> and I am a <role & institution> working on the St Paul’s Hospital team for the Perioperative Opioid Quality Improvement project.

*Just to remind you, we are doing this to find out what you think of the Thrive and/or Careteam websites and apps that you have been using since your surgery, if you think it helped you after your surgery, and what you liked or did not like about it.*

I want to let you know that if you have any medical questions regarding your health, I will not able to give medical advice. However, I can pass the information on to someone who will get back to you.

*OPTIONAL IF CONDUCTED OVER ZOOM*: I would like to remind you that we will be live-transcribing the interview. However, there will not be any directly identifying information on the transcribed document as your name will be replaced with a code number to protect your privacy.

Finally, we would like to use anonymous quotes to use in scientific abstracts, journal publications, and advertisement materials. Do you have any questions before I start?”

*Note.* *Probing questions do NOT need to be used in their entirety; they are used to gather additional insights if the participant does not cover these points in their response naturally. Aim to address all relevant probes but ensure the call is limited to 15 minutes and all questions are responded to.*

## Interview Questions:

***Case 1:*** For the patient who is in the **high-risk** category

1. Could you please describe your general experience while using the Thrive platform before surgery?
   1. Probe: Was the Thrive platform simple to use? If not, why?
   2. Probe: What functions and capabilities of the Thrive platform were useful? Alternatively, what features and capabilities should be added to the Thrive platform?
2. Could you please describe your general experience while using Careteam (e.g. in hospital/clinic, before and after surgery)?
   1. Probe: Were you satisfied with the tasks the Careteam website and app support? For example, the knowledge resources in Careteam, sharing your care plan with your family and/or friends. If not, why?
   2. Probe: Was it simple to use? If not, why?
   3. Probe: Could you find the information (e.g. care plan, your responses to questionnaires, pain management) you wanted easily?
   4. Probe: What functions and capabilities of Careteam were useful? Alternatively, what features and capabilities should be added?
3. Did using Careteam assist your post-surgical healthcare experience?
   1. Probe: For example, did you find Careteam helpful in managing your symptoms and/or general recovery? If you have had similar healthcare experiences previously, in what ways did Careteam change your recovery?
   2. Probe: Did you use the educational information surrounding your care? Did you find these helpful? If they were not available in Careteam, would you have used these materials if they were provided to you in other ways?
4. Do you have any other feedback or anything else you want to state about your experience using Thrive and/or Careteam?
   1. Probe: Did using these tools have an impact on your overall experience? Why or why not?
   2. Probe: Is this something you would recommend to your friends or family, why/why not?
5. Is there other information you would have liked to have seen in the application?
6. Were you satisfied with the care provided by the Transitional pain clinic?
7. What was positive about your experience with the Transitional Pain Clinic?
8. What could be improved about your experience with the Transitional Pain Clinic.

***Case 2:*** For the patient who is in the **low-risk** category.

1. Could you please describe your general experience while using Thrive (e.g. in hospital/clinic, before and after surgery)?
   1. Probe: Did you benefit from knowing your postoperative risk score? If not, why not?
   2. Probe: Was it simple to use? If not, why not?
   3. Probe: Could you find the information you wanted easily? Was there any information you were missing pre or post-op or difficult to get?
   4. Probe: How was your experience completing an online questionnaire compared to a paper version?
   5. Probe: What functions and capabilities of the Thrive platform were useful? Alternatively, what features and capabilities should be added to Thrive?
2. How was your overall healthcare experience in hospital and/or after returning home while using Thrive?
   1. Probe: For example, did the platform improve the recovery process (e.g. reduce pain, nausea, vomiting, anxiousness/worry)?
   2. Probe: Do you feel that you had improved access to educational information surrounding your care?
   3. Probe: What did you find useful/not useful? Did you find Thrive helped you? Why or why not?
3. Do you have any other feedback or anything else you want to state about your experience using Thrive?

# Interview Guide (Healthcare personnel version)

## Tasks to For Each Telephone Interview:

1. Ask to speak with the consented patient and have the speaker confirm.
2. Confirm now is a convenient and safe time to talk and that they consent to speak with you for approximately 10-15 minutes; otherwise re-arrange for a later date.
3. Explain the format of the brief interview (over Zoom or telephone) and that you will be using Zoom’s live transcription function or taking handwritten notes to record their answers and will ask additional clarifying questions as needed.
4. Thank them for their participation and their willingness to speak today.

## Participant introduction for each session:

“Good morning/afternoon/evening, thank you for taking the time to join me today. My name is <your name> and I am a <role & institution> working on the St Paul’s Hospital team for the Perioperative Opioid Quality Improvement project.

*Just to remind you, we are doing this to find out what you think of the Thrive and/or Careteam websites and apps that you have been using with your patients, if you think it helped their recovery process, and what you liked or did not like about it.*

*OPTIONAL IF CONDUCTED OVER ZOOM*: I would like to remind you that we will be live transcribing the interview. However, there will not be any directly identifying information on the transcribed document as your name will be replaced with a random number to protect your privacy.

“Finally, we would like to use anonymous quotes to use in scientific abstracts, journal publications, and advertisement materials. Do you have any questions before I start?”

*Note.* *Probing questions do NOT need to be used in their entirety; they are used to gather additional insights if the participant does not cover these points in their response naturally. Aim to address all relevant probes but ensure the call is limited to 15 minutes and all questions are responded to.*

## Interview Questions:

1. Do you use Thrive and/or Careteam as part of patients’ perioperative journey?
   1. *If yes: please ask the provider about both the platforms*
   2. *If no: Please ask about which platform that the provider is using*
2. How do you use Thrive currently?
   1. Probe: What do you mainly check Thrive for?
   2. Probe: How does Thrive fit in your current daily workflow?
3. How did you use Careteam? What features did you use the most?
   1. Probe: What are the main benefits of using Careteam?
   2. Probe: Would you use Careteam differently next time?
4. Could you please describe your general experience while using Thrive/Careteam (e.g. in hospital/clinic, before and after surgery)?
   1. Probe: For example, were you satisfied with the Thrive/Careteam platform?
   2. Probe: In what context did you use Thrive/Careteam and was it simple to use?
   3. Probe: Are there challenges or frustrations using Thrive/Careteam? What works well for you?
   4. Probe: Were you able to easily onboard patients using Careteam platform?
   5. Probe: What functions and capabilities of the Thrive/Careteam that you were useful? Alternatively, what features, and capabilities would you add to Thrive/Careteam in the future?
   6. Probe: What were the benefits that you and your patients received from using Thrive/Careteam?
   7. Probe: Did Thrive/Careteam meet your expectations?
5. Do you believe using the platforms improved the healthcare experience of your patients both in hospital and once they return home?
   1. Probe: To what extent do you feel it will or has improved patients’ understanding of their care
   2. Probe: Do you think using Thrive/Careteam had an impact on the patients’ recovery? (if yes, how and was it for the better or worse?)
6. Do you think the Thrive/Careteam platform has had any impact on the post-operative (opioid) requirements of your patients
   1. Probe: if yes, how, and by how much?
   2. Probe: if no, why do you think it hasn’t (or is it too early to tell)
   3. Probe: if no, do you think it has the potential to do so, and if so, do we need to change anything to realize that potential?
7. Do you have any other feedback or anything else you want to state about your experience using Thrive/Careteam?
   1. Probe: Perhaps, what could we do to improve Thrive/Careteam for future patients?
